# Smoking, immunity, and cardiovascular prognosis: a study of plasma IgE concentration in patients with acute myocardial infarction

**DOI:** 10.3389/fcvm.2023.1174081

**Published:** 2023-09-05

**Authors:** Lili Zhang, Yanrong Zhu, Xin Meng, Yifan Zhang, Qian Ren, Dong Huang, Zhong Chen

**Affiliations:** ^1^ Department of Cardiology, Shanghai Sixth People’s Hospital Affiliated to Shanghai Jiao Tong University School of Medicine, Shanghai, China; ^2^ Department of Clinical Nutrition, Shanghai Sixth People’s Hospital Affiliated to Shanghai Jiao Tong University School of Medicine, Shanghai, China; ^3^ Department of Cardiology, Shanghai Sixth People’s Hospital Fujian, Fujian, China

**Keywords:** acute myocardial infarction, atherosclerosis, smoking, cotinine, immunoglobulin E

## Abstract

**Background:**

Immunoglobulin E (IgE) is implicated in the pathogenesis of acute myocardial infarction (AMI), and smokers often exhibit elevated plasma IgE levels. However, it remains uncertain whether the role of smoking in the development and prognosis of AMI is influenced by IgE levels. This study aimed to investigate the potential contribution of IgE in mediating the association between smoking and AMI.

**Methods:**

We conducted a prospective study involving 348 consecutive patients with chest discomfort who underwent coronary angiography. Plasma cotinine, an alkaloid present in tobacco, and IgE levels were measured. The patients were followed up for mean 39-months to assess their long-term prognosis based on major adverse cardiac and cerebrovascular events (MACCE).

**Results:**

Our findings indicate that patients with AMI had higher plasma levels of cotinine and IgE. Univariate analyses demonstrated a positive association between plasma cotinine (OR* *=* *1.7, 95% CI: 1.27–2.26, *P *< 0.001) and IgE (OR* *=* *2.8, 95% CI: 1.75–4.39, *P *< 0.001) with AMI. Receiver operating characteristics analyses showed that the combined use of cotinine and IgE (AUC: 0.677) had a larger predictive performance compared to cotinine alone (AUC: 0.639) or IgE alone (AUC: 0.657), although the improvement did not reach statistical significance. Multivariable logistic regression revealed a positive association between plasma cotinine and AMI (OR* *=* *1.70, 95% CI: 1.04–2.78, *P *= 0.036). Furthermore, the inclusion of plasma IgE in the regression model led to a decrease in the OR and 95% CI of plasma cotinine (OR* *=* *1.66, 95% CI: 1.01–2.73, *P *= 0.048). Process mediation analyses showed a significant indirect effect of plasma cotinine on AMI mediated through increased plasma IgE. Kaplan–Meier analysis during a mean 39-months follow-up revealed that higher plasma levels of IgE were associated with an increased risk of MACCE following AMI (*P* = 0.047). However, in the context of the COX regression analysis, no significant correlation was observed between IgE, cotinine and AMI.

**Conclusion:**

Cotinine exhibits a positive association with AMI, wherein IgE plays a mediating role. Elevated plasma levels of IgE was positively associated with AMI and poor prognosis, which further confirms the adverse role of smoking on the incidence of AMI and prognosis. (Clinical trial registration: ChiCTR2100053000).

## Introduction

Atherosclerotic cardiovascular diseases continue to be the leading cause of morbidity and mortality worldwide (1). Acute myocardial infarction (AMI) refers to acute myocardial tissue damage resulting from coronary thrombosis and subsequent occlusion of the coronary arteries, usually triggered by the erosion or rupture of unstable atherosclerotic plaques (2). Smoking, a recognized major risk factor of mortality (3, 4), significantly contributes to cardiovascular disease (CVD) including AMI. It is associated with various mechanisms such as inflammation, immune response, platelet dysfunction, impaired endothelial function, and increased oxidative stress, which collectively contribute to the development of coronary heart disease (CHD) and AMI (5, 6). However, the precise underlying mechanism through which smoking affects the progression of AMI remains elusive.

Immunoglobulin E (IgE) plays a crucial role in the pathogenesis of allergic responses by mediating the activation of macrophages, leading to the release of inflammatory molecules such as TNF-α and IFN-γ (7). Experimental studies have indicated an increase in IgE level within atherosclerotic lesions in mice, with observed colocalization alongside plaque macrophages (7). Notably, IgE deficiency in mice has been shown to confer protection against diet-induced atherosclerosis and foam cell formation (8). Additionally, IgE has been implicated in promoting the accumulation of neutrophils in atherosclerotic lesion through mast cells activation (9). Clinical studies have reported an association between plasma IgE level and multi-vessel coronary disease, with elevated IgE levels observed in patients with AMI or unstable angina pectoris (10, 11).

Emerging evidence supports the crucial role of inflammation in the pathophysiology of smoking-induced CHD (12, 13). Prolonged exposure to cigarette smoke promotes atherosclerosis by triggering the production of pro-inflammatory cytokines and immunoglobulin, such as IL-1β and TNF-α (14, 15). Elevated levels of IgE have been found to be significantly associated with smoking (16, 17). Cotinine, the principal metabolite of nicotine in cigarettes, serves as an objective biomarker for assessing smoking exposure (18). Recent studies have demonstrated a dose-dependent relationship between cotinine-verified cigarette exposure and total serum IgE levels (19, 20), suggesting that IgE may serve as a link or mediator between smoking and CHD, including AMI, through the inflammatory immune response. However, limited data are available regarding the correlation between IgE, smoking and AMI, as well as worsened prognosis.

Given the existing knowledge gaps, our study aimed to evaluate the levels of two specific factors, cotinine and IgE, and investigate the associations among cotinine, IgE, and smoking exposure. Additionally, we conducted a prospective study of AMI patients, to assess the occurrence of major adverse cardiac and cerebrovascular events (MACCE) and explore the potential roles of smoking and IgE. Hypothetically, we propose that cotinine-verified smoking serves as a risk factor for AMI and predicts poor prognosis through an inflammatory response mediated by IgE.

## Materials and methods

### Study population

This single center study was conducted from February 2019 to June 2020. We recruited 372 hospitalized patients with suspected CHD for coronary angiography. Patients were excluded if they had previous myocardial infarction within six months (*n* = 2); history of asthma (*n* = 6), malignancies, autoimmune diseases; and treatment with anti-inflammatory drugs (*n* = 5). Patients with advanced liver disease, renal failure, and valvular heart disease were also excluded (*n* = 5). A total of 6 patients with AMI were lost during the 3-year follow-up. The study population included 260 patients with non-AMI (including 92 non-CHD patients and 168 stable CHD patients), and 88 patients diagnosed with AMI (including 55 patients with ST-segment elevation myocardial infarction and 33 patients with non-ST-segment elevation myocardial infarction). AMI patients were followed during a mean period of 39 months and categorized according to levels of IgE and cotinine (Supplementary Figure S1).

All non-CHD patients had a history of suspicious ischemic symptoms with CVD risk factors. However, they had no evidence of coronary stenosis based on coronary angiography. CHD was diagnosed if coronary angiography revealed at least one significant stenosis (defined as ≥50% luminal narrowing in the main coronary arteries or major side branches). Identification of AMI was performed following the recent consensus documents of the American Heart Association and the American College of Cardiology (21).

### Clinical parameters recorded at baseline

Patient information was recorded, including age, sex, and cardiovascular risk factors. Smoking status was categorized as smoker and nonsmoker (lifetime smoking history of fewer than 100 cigarettes). Smoking index was calculated using average number of cigarettes smoked per day multiplied by the number of years of smoking. Diagnosis of diabetes was made according to the criteria of the American Diabetes Association (fasting blood glucose concentration of ≥7 mmol/L or the use of insulin or oral hypoglycemic medications) (22). Hypertension was defined as systolic blood pressure ≥140 mmHg, diastolic blood pressure ≥90 mmHg, or the use of medications prescribed for hypertension (23). Hyperlipidemia was defined based on total fasting levels of cholesterol ≥240 mg/dl, triglycerides ≥200 mg/dl, high-density lipoprotein cholesterol (HDL-C) ≤40 mg/dl, low-density lipoprotein cholesterol (LDL-C) ≥160 mg/dl, and/or treatment for hyperlipidemia (24).

### Biochemical measurements

Blood samples were obtained from all participants while in the recumbent position via antecubital venipuncture at the time of hospital admission. The plasma concentration of high-sensitivity C-reactive protein (hs-CRP), white blood cells (WBC), uric acid, creatinine, alanine aminotransferase (ALT), asparagine transaminase (AST), glycated hemoglobin (HbA1C), N-terminal pro-brain natriuretic peptide (NT-proBNP), cardiac troponin I (cTNI), and lipid profiles, including total cholesterol, triglyceride, LDL-C, and HDL-C, were measured according to standard laboratory protocols. Plasma levels of cotinine and IgE were determined by enzyme-linked immunosorbent assay (ELISA), according to the manufacturer's protocols (CO096D, CALBIOTECH, EI Cajon, CA, USA; SX05002, Senxiong, Shanghai, China).

### Doppler echocardiography

All participants underwent transthoracic two-dimensional and M-mode echocardiography following recommendations provided by the American Society of Echocardiography (25). Left ventricular ejection fraction (LVEF) was calculated using the modified Simpson method.

### Follow-up

Clinical follow-up was conducted using a structured questionnaire of the patient's inpatient medical records, regular outpatient visits, and telephone interview. 88 patients with AMI were followed up for 30–46 months (average 39 months). Six patients were lost during follow-up (Supplementary Figure S1). The primary clinical endpoint was MACCE during follow-up and defined as all-cause death, target vessel revascularization, myocardial infarction, unstable angina pectoris requiring re-hospitalization, heart failure, stroke, or transient cerebral ischemia. Every 3 months, an interviewer contacted each participant or his/her family member about MACCE. All potential endpoint events were adjudicated by an assessment committee whose members were blind to patients’ characteristics.

### Statistical analysis

For the continuous variables, data were expressed as the median with inter-quartile ranges. Differences in data were evaluated using Mann–Whitney *U*-test for comparisons between groups. Categorical variables were summarized as frequencies (percentages) and compared by chi-square test. Partial correlation test was used to assess the correlation between two variables. To determine the association between independent variables and AMI, logistic multivariate regression analysis was performed for indicators with *P* < 0.05 in univariate analysis to calculate odds ratio (OR) and 95% confidence interval (95%Cl). Cotinine, IgE, and NT-proBNP with larger degree of dispersion were analyzed as log-transformed normally distributed variables. Receiver-operating characteristic (ROC) curves were plotted to determine the cotinine and cotinine with IgE for detecting AMI. Using the approach by Hayes and Preacher (26), the direct and indirect effect sizes of the association between cotinine and incidence of AMI were determined by using IgE as mediators. Kaplan–Meier survival curves were conducted to estimate the survival status of patients with cotinine and IgE within mean 39 months after AMI. Statistical analysis was performed using SPSS Statistical software, version 22.0 (IBM, Chicago, IL, USA). The mediation analyses were performed using Process version 3.5 added on to SPSS. Two-sided *P*-values of <0.05 were considered statistically significant. The ROC curve and Kaplan–Meier survival curves were plotted using GraghPad Prism 7.0 (GraphPad Prism Software Inc., San Diego, CA).

## Results

### Clinical characteristics of the study participants

In the current study, we included 348 study participants (209 men and 139 women; mean age 66.64 ± 11.91 years) of which 260 patients were diagnosed with non-AMI, and 88 patients were diagnosed with AMI. The baseline clinical characteristics in the non-AMI and AMI patients are presented in Table 1. Patients in the AMI group were mainly men and had a higher exposure to tobacco smoke (37.69% vs. 48.86%, *P* = 0.03), higher smoking index (0 [0, 400] vs. 20 [0, 600], *P *= 0.01). There were no significant differences in age and history of hypertension, diabetes, or hyperlipidemia between the two groups. At baseline, hs-CRP, WBC, creatinine, cTNI, NT-proBNP among AMI group were significantly higher, whereas HDL-C and LVEF were lower compared with that in the non-AMI group. In addition, the plasma levels of cotinine (1.28 [0.82, 2.75] vs. 2.08 [1.10, 51.30], *P *< 0.001) and IgE (54.97 [27.17, 122.46] vs. 110.63 [53.82, 351.38], *P *< 0.001) were significantly higher in the AMI group.

**Table 1:** Clinical characteristics of study patients at baseline.

	non-AMI (*n* = 260)	AMI (*n* = 88)	*P* value
Men, *n* (%)	140 (53.85)	69 (78.41)	<0.001
Age, year	67 (59, 75)	67 (57.25, 78)	0.928
Smoking index	0 (0, 400)	20 (0, 600)	0.001
hs-CRP, mg/L	0.65 (0.53, 2.13)	2.95 (0.74, 17.34)	<0.001
WBC, *10^9^/L	6.35 (5.28, 7.52)	8.65 (6.57, 11.65)	<0.001
TC, mmol/L	4.37 (3.71, 5.01)	4.48 (3.71, 5.43)	0.302
TG, mmol/L	1.35 (1.09, 1.84)	1.54 (1.10, 2.01)	0.177
HDL-C, mmol/L	1.07 (0.92, 1.25)	1.01 (0.84, 1.14)	<0.001
LDL-C, mmol/L	2.67 (2.2, 3.11)	2.74 (2.28, 3.52)	0.109
HbA1c, %	6.0 (5.6, 6.6)	6.2 (5.6, 6.7)	0.351
Serum creatinine, μmol/L	69 (55.5, 80)	78 (63, 92)	<0.001
cTNI, ng/ml	3.0 (0.02, 5.41)	4.3 (1.10, 13.77)	<0.001
NT-proBNP, pg/ml	116.5 (45.60, 503.53)	1101 (188.25, 3331.50)	<0.001
LVEF, %	60 (57, 64)	58 (55, 61)	<0.001
Cotinine, ng/ml	1.28 (0.82, 2.75)	2.08 (1.10, 51.30)	<0.001
IgE, ng/ml	54.97 (27.17, 122.46)	110.63 (53.82, 351.38)	<0.001
History, *n* (%)			
Current smoking	98 (37.69)	47 (53.41)	0.012
Hypertension	177 (68.08)	51 (57.95)	0.093
Diabetes	61 (23.46)	25 (28.41)	0.391
Hyperlipidemia	20 (7.69)	11 (12.50)	0.194

AMI, acute myocardial infarction; hs-CRP, high sensitivity C-reactive protein; WBC, white blood cells; TC, total cholesterol; TG, triglyceride; LDL-C, low-density lipoprotein cholesterol; HDL-C, high-density lipoprotein cholesterol; HbA1c, glycated hemoglobin; cTNI, cardiac troponin I; NT-proBNP, N-terminal pro-brain natriuretic peptide; LVEF, left ventricular ejection fraction.

### The characteristics of cotinine and IgE in patients with a history of smoking

**Figure 1:**
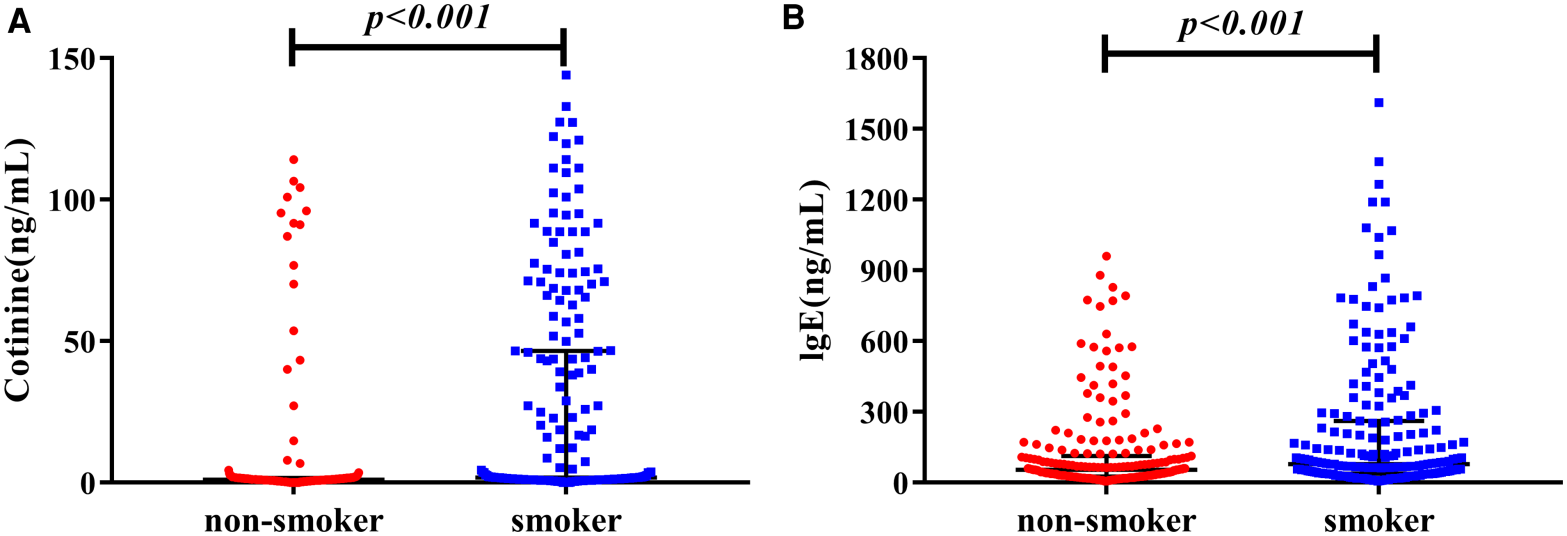
The distribution nof cotinine and IgE in non-smokers vs. smokers. (**A**) Shows plasma level of cotinine, and (**B**) shows IgE concentration in plasma.

Because cotinine is the main metabolite of nicotine and smoking might induce the elevation of plasma IgE, we compared the difference of cotinine, IgE, and other parameters between smokers and non-smokers (Supplementary Table S1). Compared to non-smokers, smokers were younger (*P *< 0.001), had higher plasma creatinine (*P *< 0.001), cotinine (12.27 [1.33, 67.09] vs. 1.07 [0.66, 1.65], *P *< 0.001) and IgE (87.37 [38.62, 288.78] vs. 55.09 [26.20, 113.06], *P *< 0.001) (Figure 1). Furthermore, we investigated the correlations between plasma cotinine, and plasma IgE levels. After adjusting for hs-CRP, positively week correlations were found between plasma cotinine and plasma IgE levels (*r* = 0.174, *P *= 0.002).

### IgE as a mediator of the association between cotinine and AMI

**Figure 2:**
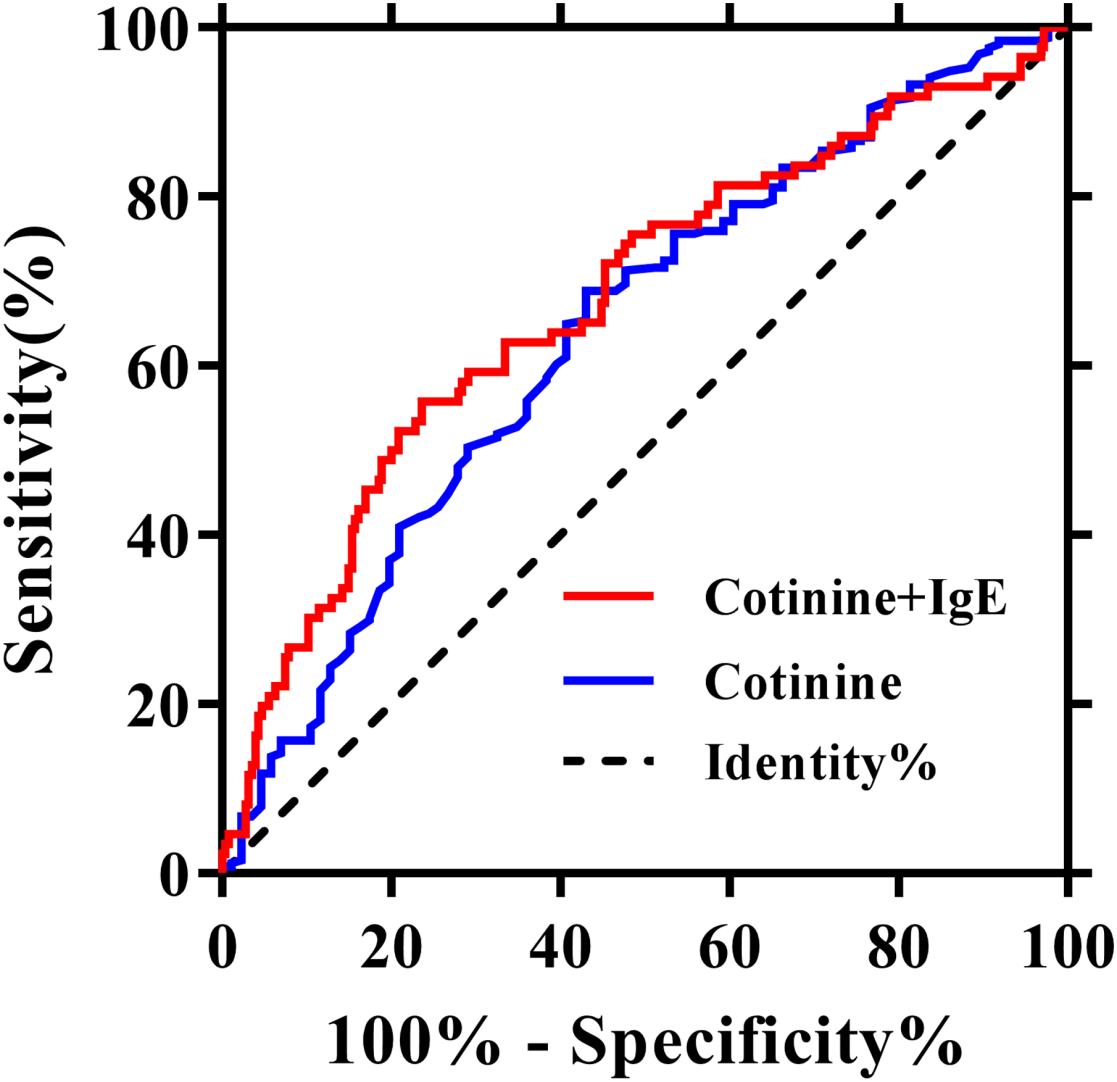
Receiver operating characteristics analyses using cotinine (log 10, ng/ml) with or without IgE (log 10, ng/ml) as predictors of myocardial infarction.

To assess the association between cotinine and IgE and its effect on the risk of AMI, we used univariate logistic regression analyses. The results indicated a positive association between plasma cotinine (log 10) (OR = 1.7, 95% CI: 1.27–2.26, *P* < 0.001) and AMI. Similarly, there was a positive association between plasma IgE (log 10) (OR = 2.8, 95% CI: 1.75–4.39, *P* < 0.001) and AMI. To investigate whether cotinine and IgE could provide better prediction of AMI compared with plasma cotinine alone, we performed ROC analyses. The results showed that the area under the curve (AUC) for IgE was 0.657 (95% CI: 0.590–0.725) (Supplementary Figure S1). As shown in Figure 2, AUC for cotinine was 0.639 (95% CI: 0.571–0.708), whereas AUC for cotinine combined with IgE was 0.677 (95% CI: 0.608–0.745). These data indicated that the combined AUROC of cotinine and IgE was higher than that of cotinine alone, although the difference did not reach statistical significance (Table 2).

**Table 2:** AUROC differences between cotinine + IgE with cotinine and IgE alone.

A	B	AUROC (A)	AUROC (B)	*P* value
IgE (log 10, ng/ml)	Cotinine (log 10, ng/ml)	0.657	0.639	0.560
Cotinine (log 10, ng/ml)	Cotinine + IgE	0.639	0.677	0.320

As smoking exposure increases plasma cotinine and activates inflammation and expression of IgE, we investigated whether the elevation of IgE might explain the association between cotinine and AMI by analyzing the association between plasma cotinine (log 10) and incidence of AMI with or without IgE (log 10). By univariate analyses, we confirmed that sex, WBC, HDL-C, hs-CRP, LVEF, cTNI, and NT-proBNP (log 10) were significantly positively associated with AMI (Supplementary Table S2). In multivariable analyses, only hs-CRP, WBC, cTNI, NT-proBNP (log 10), and cotinine (log 10) (OR = 1.70, 95% CI: 1.04–2.78, *P *= 0.036) remained statistically significant. When IgE (log 10) was added into the multivariable analyses model, a decrease in the association of cotinine (OR = 1.66, 95% CI: 1.01–2.73, *P *= 0.048) with incident AMI was observed (Table 3). However, there is no significant association between IgE (log 10) and AMI.

**Table 3:** Logistic regression analysis of the correlation between cotinine and AMI with or without IgE.

	Model 1 (*n* = 348)		Model 2 (*n* = 348)	
	OR (95% CI)	*P* value	OR (95% CI)	*P* value
Male	0.40 (0.16–1.02)	0.056	0.40 (0.15–1.02)	0.054
hs-CRP	1.05 (1.01–1.09)	<0.001	1.05 (1.00–1.09)	0.043
WBC	1.61 (1.32–1.96)	<0.001	1.60 (1.32–1.95)	<0.001
HDL-C	0.40 (0.08–1.97)	0.261	0.41 (0.08–1.99)	0.267
Serum creatinine	1.01 (0.99–1.03)	0.442	1.01 (0.99–1.02)	0.474
LVEF	0.96 (0.90–1.03)	0.963	0.96 (0.90–1.03)	0.285
cTNI	1.15 (1.06–1.26)	0.001	1.15 (1.05–1.25)	0.003
NT-proBNP (log 10)	2.82 (1.48–5.36)	0.002	2.83 (1.48–5.41)	0.002
Cotinine (log 10)	1.70 (1.04–2.78)	0.036	1.66 (1.01–2.73)	0.048
IgE (log 10)			1.24 (0.62–2.51)	0.543

hs-CRP, high sensitivity C-reactive protein; WBC, white blood cell; HDL-C, high-density lipoprotein cholesterol; cTNI, cardiac troponin I; NT-proBNP, N-terminal pro-brain natriuretic peptide; LVEF, left ventricular ejection fraction, and OR, odds ratio.

To evaluate whether the reduced odds ratio is indeed due to increased IgE, we further performed mediation analyses using the statistical approach by Hayes and Preacher (26). By this method, we could estimate the significance and size of indirect effect of cotinine on AMI carried by elevated plasma IgE. As shown in Table 4, the direct effect of plasma cotinine on AMI was 0.34 (95% CI: 0.04–0.65), whereas its indirect effect was 0.08 (95% CI: 0.02–0.17). The proportion mediated by IgE in the effect size of cotinine on AMI was 0.24.

**Table 4:** Direct and indirect effect sizes of cotinine for AMI as mediated by IgE.

	Effect	SE	*P*	95% CI	
				LLCI	ULCI
Direct effect	0.343	0.154	0.026	0.041	0.645
Indirect effect	0.083	0.039		0.018	0.170

LLCI, low limit confidence interval; ULCI, upper limit confidence interval.

### Survival analysis

**Figure 3:**
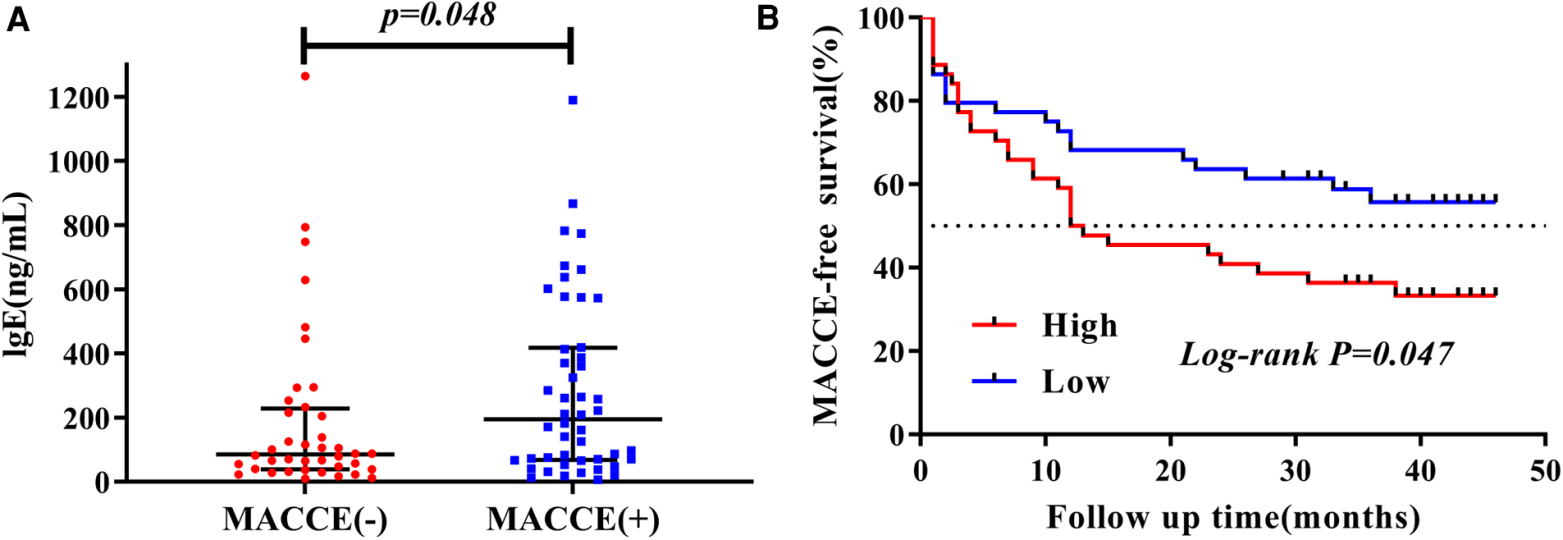
The characteristic of plasma IgE concentration during mean 39-months follow-up. (**A**) Distribution of plasma IgE in MACCE (−) vs. MACCE (+). (**B**) Kaplan–Meier survival by elevated plasma IgE.

After a median follow-up of 39 months, a total of 48 (54.5%) AMI patients suffered from MACCE. Patients with AMI were divided into two groups based on MACCE, namely MACCE (+) and MACCE (−) groups. We compared the baseline characteristics between the two groups, and found that patients in the MACCE (−) group were significantly younger than those in the MACCE (+) group (61.05 ± 13.93 vs. 70.42 ± 12.21, *P *= 0.001), and plasma IgE (84.88 [38.09, 227.55] vs. 194.49 [67.76, 417.71], *P *= 0.048), and HbA1c (6.0 [5.30, 6.30] vs. 6.40 [5.90, 7.70], *P *= 0.002) in MACCE (+) group were higher compared with that in the MACCE (−) group (Figure 3A). However, no significant differences occurred in plasma cotinine (2.88 [1.14, 51.24] vs. 1.89 [1.10, 54.30], *P *= 0.798) and other clinical markers (Supplementary Table S3).

Furthermore, patients with AMI were divided into high (Q3–Q4) and low (Q1–Q2) level groups based on plasma cotinine and IgE quartiles. The Kaplan–Meier curve depicted the follow-up without MACCE (MACCE-free) survival by comparing high and low levels of cotinine and IgE in AMI patients, followed by comparison of survival curves using the log-rank test. As presented in Figure 3B, Kaplan–Meier survival curves showed that AMI patients with higher plasma IgE had reduced MACCE-free survival time (*P *= 0.047). However, no difference was found for survival time between high and low levels of plasma cotinine (*P *= 0.62, Supplementary Figure S2).

We further conducted multivariate COX proportional hazard regression analysis to identify risk factors for MACCE. However, after adjustment for confounders, the results showed that only age was significantly associated with MACCE, while IgE and cotinine did not show any significant correlation with MACCE (Table 5).

**Table 5:** Multivariate cox proportional hazards regression analyses for the risk factors of MACCE during follow-up among patients with AMI.

	HR	95% CI	*P* value
Age, year	1.04	1.01–1.07	0.004
Cotinine (log 10)	1.22	0.77–1.96	0.399
IgE (log 10)	1.53	0.84–2.81	0.167

## Discussion

In the present study, we observed a significant elevation in the concentrations of both plasma cotinine and IgE in patients with AMI. Both elevations in plasma cotinine and IgE were associated with AMI, and the predictive performance of cotinine combined with IgE was better than that of either alone. Importantly, our data suggest that the impact of cotinine on AMI is partly mediated by an increase in plasma IgE concentration. Additionally, plasma IgE, not cotinine, was significantly associated with MACCE events within a mean 39-months follow-up after AMI.

Elevated plasma concentration of IgE has been investigated as a potential risk factor for CVDs (11, 27). Meanwhile, studies have shown that circulating IgE levels in patients with atherosclerosis are associated with plaque instability and severity of CHD (7, 10). Immunologic mechanisms play a major role in the pathogenesis of coronary plaque instability, leading to AMI (28). IgE, as an important regulator of allergic reactions, binds to high and low affinity FcεRI and FcεRII receptors on the mast and macrophage cell surface. Research by Shi et al. (7, 8) revealed that IgE could promote the formation of atherosclerosis by stimulating cell apoptosis and cytokine expression, influencing macrophage polarization, and facilitating foam cell formation. Consistent with these findings, our study demonstrates a significant increase in plasma IgE levels in patients with AMI, suggesting its potential as a predictive marker for AMI based on AUC analyses.

Smoking disturbs immune homeostasis by modulating immune-regulatory activities, leading to various pathogenic mechanisms, including increased oxidative stress, reactive oxygen species (ROS), and the activation of T and B cell proliferation, which subsequently enhance the expression of pro-inflammatory markers (29). Studies have consistently reported elevated levels of circulating IgE in smokers (19, 30, 31). Furthermore, previous research has demonstrated that IgE plays a role in amplifying the immune-inflammatory response associated with smoking. It can induce neutrophil activation, mast cell activation, further exacerbating the inflammatory and cytokine cascade (32, 33). In this study, we employed plasma cotinine measurement, widely recognized as a reliable biomarker of tobacco exposure (18, 34), to ascertain the smoking status and self-reported cigarettes per day. Consistent with previous findings (19, 20), our data revealed higher plasma cotinine and IgE levels in patients with a smoking history. Additionally, we identified an intrinsic association between cotinine and IgE levels, suggesting that smoking may partially induce an inflammatory response through plasma IgE. Notably, as the major metabolite of nicotine, cotinine offers a more accurate estimation of smoking status.

A higher proportion of patients with AMI were found to have a history of smoking and elevated plasma cotinine levels, which is consistent with previous reports (35, 36). Based on these findings, we further investigated the potential mediation effect of IgE in the association between plasma cotinine and AMI using mediation analyses. Our data revealed that the association between plasma cotinine and AMI was markedly attenuated when adjusting for plasma IgE concentration. Specifically, the positive association between plasma cotinine and AMI decreased from 1.70 to 1.66 after incorporating IgE into the model, suggesting that IgE acts as a mediator in the association between plasma cotinine and AMI. Through Hayes and Preacher's mediation analysis (26), we estimated that approximately 24% of the effect of cotinine on AMI was mediated by IgE. These findings confirmed that the observed positive correlation between AMI and smoking, as indicated by plasma cotinine, is partly mediated by the elevation of plasma IgE concentration.

**Figure 4:**
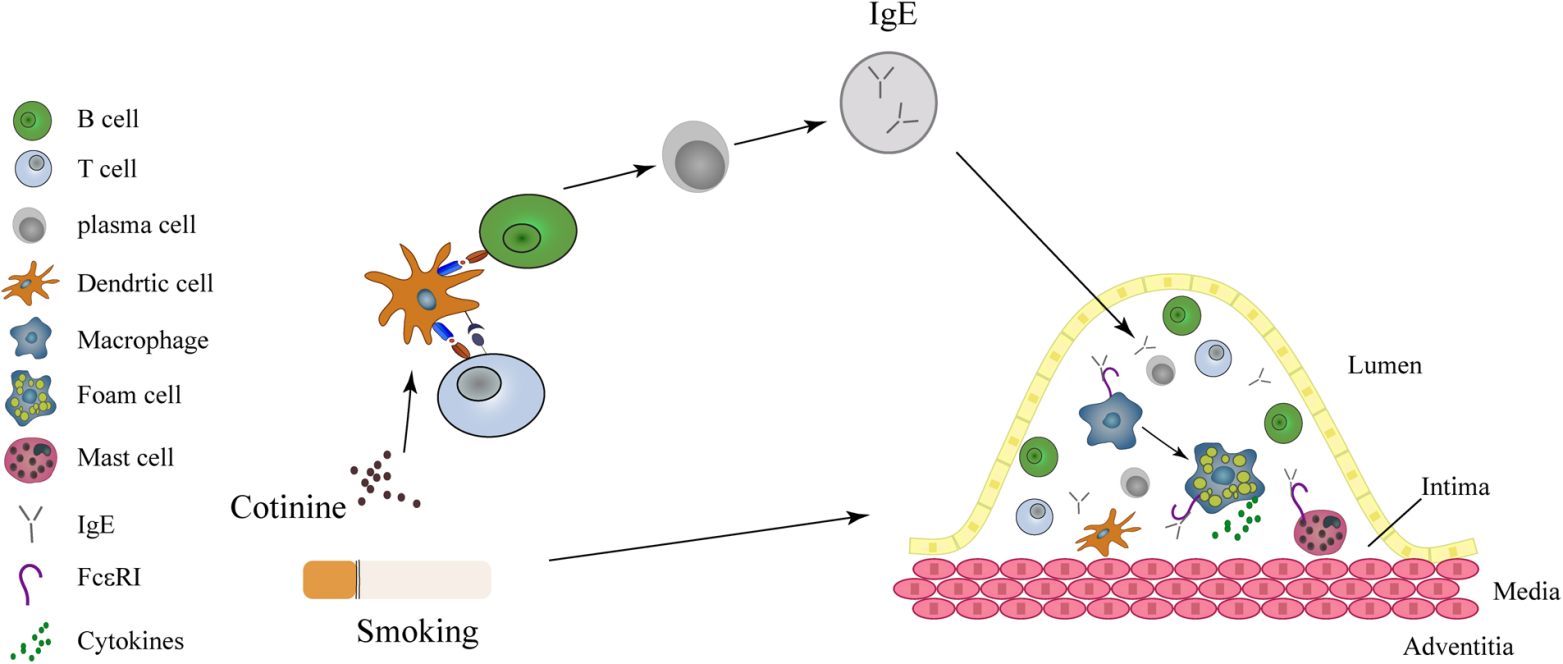
IgE acts as a mediator in the link between smoking and myocardial infarction. Smoking causes to the release of cotinine into the plasma, subsequently activating the T and B lymphocytes, resulting in the release of IgE. Within the intima, IgE activates macrophages and mast cells through FcεRI and FcεRII receptors. Both macrophages and mast cells contribute to the formation of foam cells and the instability of coronary atheromatous plaques.

Despite extensive clinical, experimental and epidemiological investigations on the association between smoking and AMI, the underlying mechanisms are still subject to ongoing scrutiny (37, 38). Smoking has been shown to activate neutrophils, T and B lymphocytes, and induce endotoxin exposure, all of which can contribute to elevated IgE levels and subsequent inflammatory response (32, 39, 40). Elevated IgE has been implicated in the pathogenesis of atherosclerosis and AMI through its activation of macrophages and mast cells, leading to cytokine secretion, foam cell formation, and the expression of IL-17/22 (7, 8, 33, 41, 42) (Figure 4). However, the specific mechanism by which smoking induces elevated IgE levels and exacerbates atherosclerosis and plaque instability requires further investigation.

Smoking is recognized as a significant determinant of cardiovascular morbidity and mortality, particularly among men worldwide and women in developed countries (43). The term “smoker's paradox” has been used to describe the controversial notion that smoking might improve cardiovascular outcomes post-AMI (44, 45). However, conflicting evidence has emerged, refuting these paradoxical findings (46–48). Our data, assessed through Kaplan–Meier analysis, indicate no difference in plasma cotinine levels between patients with or without MACCE. Furthermore, in our Cox regression analysis, both cotinine and IgE did not show any significant correlation with MACCE events after AMI. These findings may be influenced by various factors, including the cross-sectional nature of our study, which limited our ability to establish causation between plasma cotinine, plasma IgE and AMI. It is noteworthy that most patients tended to quit smoking after experiencing an AMI event, and some smokers had their infarction at an earlier age compared to non-smokers. Importantly, our study reveals that AMI patients with high plasma IgE concentration exhibit a higher incidence (*P *= 0.048) of MACCE. Both AMI and smoking can trigger immune-inflammatory responses that lead to IgE release. Furthermore, IgE not only plays a role in the acute phase of AMI, but also contributes to the process of ventricular remodeling (27, 49). Consistent with our findings, several studies have independently associated IgE with cardiovascular events and identified it as a potential risk factor for increased cardiovascular mortality (7, 49, 50). Therefore, our findings suggest that IgE may serve as a mediator in the association between smoking and AMI. Furthermore, elevated IgE levels could potentially be a risk factor for MACCE following AMI. IgE might also serve as a valuable marker for assessing the efficacy of anti-inflammatory interventions. Additionally, targeting IgE could offer an alternative therapeutic approach to prevent smoking induced AMI and improve subsequent prognosis. Our study also identified several clinical variables that were positively associated with AMI, including hs-CRP, WBC, cTNI, and NT-proBNP, consistent with findings from previous studies (51–54). While hs-CRP and WBC partially reflect the inflammatory response during AMI occurrence, we investigated the potential role of IgE as an immune-inflammatory marker in this context. Surprisingly, our logistic regression analysis demonstrated that the inclusion of IgE (log 10) did not result in significant changes in the OR values of hs-CRP and WBC. This finding suggests that IgE may not play a substantial role in the inflammatory response associated with hs-CRP and WBC in the context of AMI. On the other hand, cTNI and NT-proBNP, as direct indicators of myocardial ischemic necrosis, have been extensively studied and have consistently shown a strong correlation with AMI (53, 54). The robust association of these biomarkers with AMI further reinforces the reliability of our data.

These are several limitations to the present study. Firstly, the cross-sectional nature of our analysis limits our ability to establish causation between plasma cotinine, plasma IgE and AMI. It is advisable to consider the temporal relationship between plasma cotinine and IgE levels in future analyses. Additionally, it is important to assess the smoking status after AMI to better understand its impact on the observed associations. Secondly, the relatively small sample size and single-center recruitment of participants may affect the generalizability of our findings. However, our study benefits from a well-designed research protocol and utilizes established methods, with the advantage of comprehensive evaluation through coronary angiography, laboratory testing, and clinical assessment, ensuring enrollment of confirmed AMI and non-AMI subjects. Although AMI can be clinically classified into ST-segment elevation myocardial infarction (STEMI) and non-ST-segment elevation myocardial infarction (NSTEMI), both conditions are considered acute coronary syndromes characterized by acute myocardial injury due to coronary artery ischemia. As IgE is involved in acute inflammatory responses, it is plausible that there may be no significant differences between the two groups regarding acute myocardial injury and inflammatory response. Finally, the cotinine-based correction introduces potential bias as cotinine levels can be influenced by other factors, which may in turn affect a minority of the participants. Further studies with larger sample sizes are warranted to validate our findings and elucidate the underlying biological mechanisms.

## Conclusion

Our study demonstrates that elevated plasma cotinine and IgE concentrations are associated with AMI. IgE may partially explain the increased incidence of AMI in smokers as indicated by cotinine measurement. Additionally, IgE shows promise as a predictor of MACCE after AMI. These findings support the role of elevated IgE as a mediator in the pathogenesis of smoking-related cardiovascular disease. Targeting the IgE pathway and its associated inflammatory mechanisms may hold promise for preventing fatal cardiovascular events among smokers.

## Data Availability

The raw data supporting the conclusions of this article will be made available by the authors, without undue reservation.
