# Supplementary material for: Smoking, immunity, and cardiovascular prognosis: a study of plasma IgE concentration in patients with acute myocardial infarction
Source: Front Cardiovasc Med. 2023 Sep 5;10:1174081. doi: 10.3389/fcvm.2023.1174081 (PMC10508960; doi:10.3389/fcvm.2023.1174081)
Supplement: Supplementary file 1 [file Datasheet1.docx]

**Data Supplement**

**Table S1** Clinical data of participants grouped by history of smoking

|  | non-smoking (n = 203) | smoking (n = 145) | *P* value |
| --- | --- | --- | --- |
| Male, n (%) | 69 (34.0) | 140 (96.56) | < 0.001 |
| Age, y | 69.0 (61.0, 79.0) | 65 (57.0, 71.0) | < 0.001 |
| hs-CRP, mg/L | 0.79 (0.53, 3.14) | 0.77 (0.56, 4.41) | 0.285 |
| WBC, *10^9^/L | 6.51 (5.56, 7.94) | 7.01 (5.55, 8.81) | 0.141 |
| TC, mmol/L | 4.43 (3.72, 5.26) | 4.33 (3.68, 5.14) | 0.290 |
| TG, mmol/L | 1.36 (1.08, 1.82) | 1.42 (1.13, 2.05) | 0.173 |
| HDL-C, mmol/L | 1.11 (0.93, 1.30) | 0.99 (0.85, 1.13) | < 0.001 |
| LDL-C, mmol/L | 2.69 (2.20, 3.30) | 2.69 (2.28, 3.32) | 0.982 |
| HbA1c, % | 6.0 (5.6, 6.63) | 6.10 (5.50, 6.60) | 0.809 |
| Serum creatinine, μmol/L | 65.0 (50.0, 81.0) | 73.0 (64.0, 86.0) | < 0.001 |
| cTNI, ng/mL | 3.0 (0.08, 6.0) | 3.50 (0.03, 6.0) | 0.891 |
| NT-proBNP, pg/mL | 186.70 (64.09, 1387.0) | 145.60 (48.44, 544.50) | 0.073 |
| LVEF, % | 60.0 (56.5, 63.0) | 60.0 (57.0, 62.0) | 0.694 |
| Cotinine, ng/mL | 1.07 (0.66, 1.65) | 12.27 (1.33, 67.09) | < 0.001 |
| IgE, ng/mL | 55.09 (26.20, 113.06) | 87.37 (38.62, 288.78) | < 0.001 |

hs-CRP, high sensitivity C-reactive protein; WBC, white blood cell; TC, total cholesterol; TG, triglyceride; LDL-C, low-density lipoprotein cholesterol; HDL-C, high-density lipoprotein cholesterol; HbA1c, glycated hemoglobin; cTNI, cardiac troponin I; NT-proBNP, N-terminal pro-brain natriuretic peptide; LVEF, left ventricular ejection fraction.

**Table S2** Univariate logistic regression analysis of the correlation between specific markers, risk factors, and AMI.

| **Variable** | **OR (95% CI)** | ***P* value** |
| --- | --- | --- |
| Male | 0.32 (0.18-0.56) | < 0.001 |
| hs-CRP | 1.08 (1.05-1.11) | < 0.001 |
| WBC | 1.64 (1.43-1.87) | < 0.001 |
| HDL-C | 0.34 (0.13-0.91) | 0.031 |
| Serum creatinine | 1.01 (1.00-1.01) | 0.060 |
| LVEF | 0.94 (0.90-0.97) | 0.001 |
| cTNI | 1.15 (1.08-1.21) | < 0.001 |
| NT-proBNP (log10) | 3.10 (2.18-4.40) | < 0.001 |

hs-CRP, high sensitivity C-reactive protein; WBC, white blood cell; HDL-C, high-density lipoprotein cholesterol; cTNI, cardiac troponin I; NT-proBNP, N-terminal pro-brain natriuretic peptide; LVEF, left ventricular ejection fraction.

**Table S3** Clinical data of AMI patients with or without MACCE

|  | MACCE (-) (n = 40) | MACCE (+) (n = 48) | *P* value |
| --- | --- | --- | --- |
| Male, n (%) | 34 (85.0%) | 35 (72.92%) | 0.201 |
| Age, y | 62.50 (51.0, 71.75) | 71.50 (63.0, 80.75) | 0.002 |
| hs-CRP, mg/L | 2.61 (0.71, 16.61) | 3.20 (0.74, 24.41) | 0.895 |
| WBC, *10^9^/L | 8.72 (6.64, 11.75) | 8.49 (6.45, 11.43) | 0.837 |
| TC, mmol/L | 4.46 (3.75, 5.52) | 4.50 (3.44, 5.37) | 0.366 |
| TG, mmol/L | 1.6 (1.15, 2.04) | 1.48 (0.93, 2.01) | 0.483 |
| HDL-C, mmol/L | 1.02 (0.84, 1.12) | 1.02 (0.84, 1.22) | 0.785 |
| LDL-C, mmol/L | 2.81 (2.39, 3.69) | 2.72 (2.04, 3.52) | 0.120 |
| HbA1c, % | 6.0 (5.30, 6.30) | 6.40 (5.90, 7.70) | 0.002 |
| Serum creatinine, μmol/L | 78.5 (67.0, 91.5) | 75.0 (58.0, 103.0) | 0.772 |
| cTNI, ng/mL | 3.49 (1.11, 12.26) | 4.76 (0.82, 14.71) | 0.968 |
| NT-proBNP, pg/mL | 420.35 (106.73, 2329.0) | 1408.0 (408.6, 5393.0) | 0.057 |
| LVEF, % | 59.0 (55.0, 61.75) | 58.0 (52.0, 61.0) | 0.293 |
| Cotinine, ng/mL | 2.88 (1.14, 51.24) | 1.89 (1.10, 54.30) | 0.798 |
| IgE, ng/mL | 84.88 (38.09, 227.55) | 194.49 (67.76, 417.71) | 0.048 |

hs-CRP, high sensitivity C-reactive protein; WBC, white blood cell; TC, total cholesterol; TG, triglyceride; LDL-C, low-density lipoprotein cholesterol; HDL-C, high-density lipoprotein cholesterol; HbA1c, glycated hemoglobin; cTNI, cardiac troponin I; NT-proBNP, N-terminal pro-brain natriuretic peptide; LVEF, left ventricular ejection fraction.


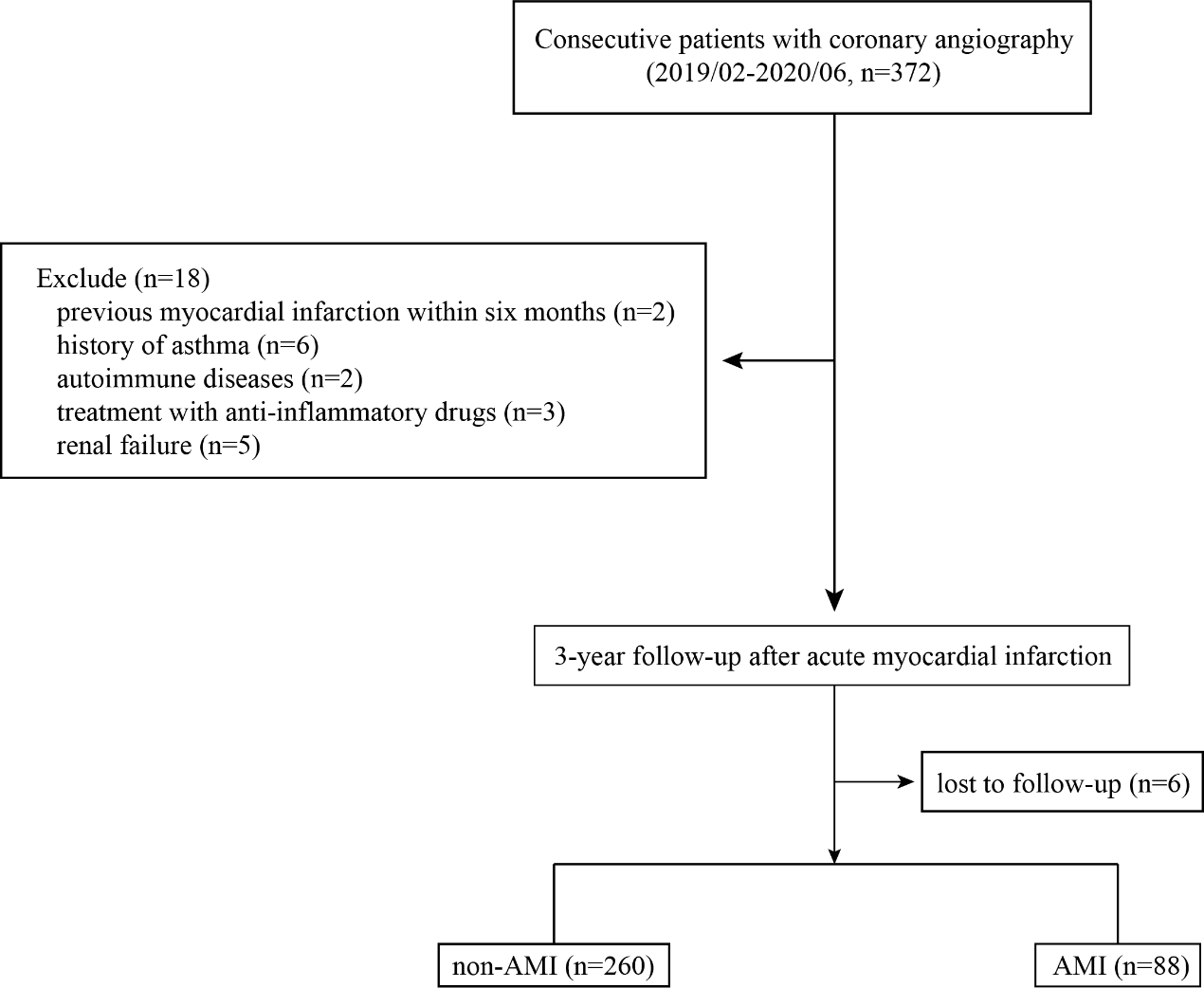


**Figure S1** Flowchart showing the inclusion of study participants.


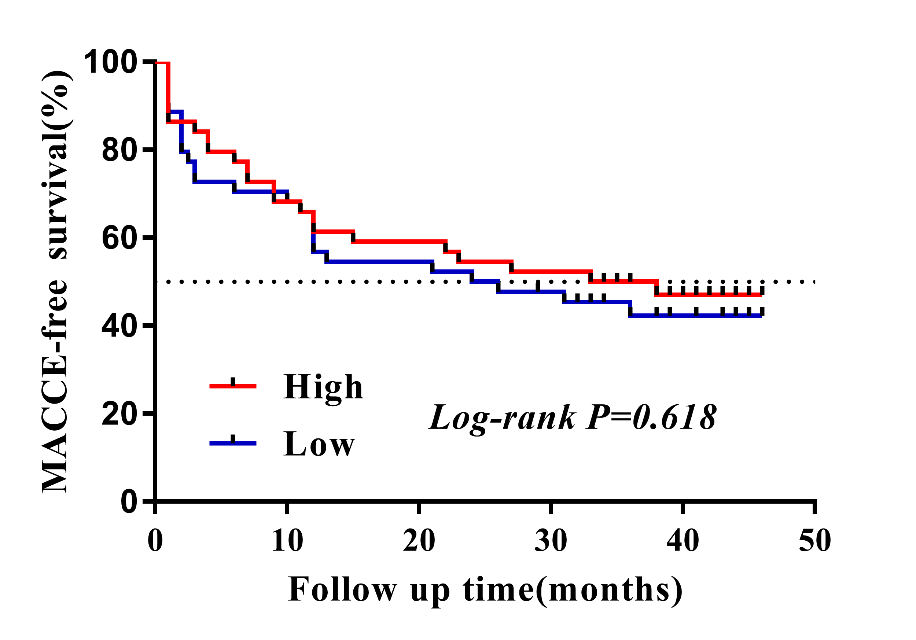


**Figure S2.** Kaplan-Meier survival by elevated plasma cotinine.
